# Predictive models of sarcopenia based on inflammation and pyroptosis-related genes

**DOI:** 10.3389/fgene.2024.1491577

**Published:** 2024-12-24

**Authors:** Xiaoqing Li, Cheng Wu, Xiang Lu, Li Wang

**Affiliations:** ^1^ Department of Geriatrics, Sir Run Run Hospital, Nanjing Medical University, Nanjing, Jiangsu, China; ^2^ Department of Geriatrics, The First Affiliated Hospital of Soochow University, Suzhou, Jiangsu, China

**Keywords:** inflammation and pyroptosis-related genes, sarcopenia, LASSO, nomogram model, predictive model

## Abstract

**Background:**

Sarcopenia is a prevalent condition associated with aging. Inflammation and pyroptosis significantly contribute to sarcopenia.

**Methods:**

Two sarcopenia-related datasets (GSE111016 and GSE167186) were obtained from the Gene Expression Omnibus (GEO), followed by batch effect removal post-merger. The “limma” R package was utilized to identify differentially expressed genes (DEGs). Subsequently, LASSO analysis was conducted on inflammation and pyroptosis-related genes (IPRGs), resulting in the identification of six hub IPRGs. A novel skeletal muscle aging model was developed and validated using an independent dataset. Additionally, Gene Ontology (GO) enrichment analysis was performed on DEGs, along with Kyoto Encyclopedia of Genes and Genomes (KEGG) pathway analysis and gene set enrichment analysis (GSEA). ssGSEA was employed to assess differences in immune cell proportions between healthy muscle groups in older versus younger adults. The expression levels of the six core IPRGs were quantified via qRT-PCR.

**Results:**

A total of 44 elderly samples and 68 young healthy samples were analyzed for DEGs. Compared to young healthy muscle tissue, T cell infiltration levels in aged muscle tissue were significantly reduced, while mast cell and monocyte infiltration levels were relatively elevated. A new diagnostic screening model for sarcopenia based on the six IPRGs demonstrated high predictive efficiency (AUC = 0.871). qRT-PCR results indicated that the expression trends of these six IPRGs aligned with those observed in the database.

**Conclusion:**

Six biomarkers—BTG2, FOXO3, AQP9, GPC3, CYCS, and SCN1B—were identified alongside a diagnostic model that offers a novel approach for early diagnosis of sarcopenia.

## Introduction

Sarcopenia is a disease characterized by the gradual loss of skeletal muscle mass, strength, and function, typically associated with aging (Cruz-Jentoft et al., 2019). This common age-related condition significantly impacts an individual’s physical health and overall quality of life (Cruz-Jentoft and Sayer, 2019). Sarcopenia becomes more prevalent with advancing age, with its incidence rising significantly among the elderly population. It affects both men and women, but its prevalence is particularly high among older adults and individuals with certain underlying health conditions (Pascual-Fernández et al., 2020). Therefore, identifying new biomarkers and uncovering immune mechanisms are crucial for prevention and treatment.

Inflammation is defined as a series of tissue responses triggered by injury, which is closely associated with various diseases (Franceschi and Campisi, 2014). Numerous studies have demonstrated that inflammation ultimately influences the mass, strength, and function of skeletal muscle by regulating protein synthesis and degradation within these muscles (Franceschi and Campisi, 2014; Zembron-Lacny et al., 2019). Tumor necrosis factor α (TNF-α) plays a pivotal role in the degradation of muscle proteins via the nuclear factor-κB (NF-κB) signaling pathway (Hirata et al., 2022). Interleukin 6 (IL-6)(IL-6), a pro-inflammatory cytokine, mediates processes that lead to either reduction or stabilization of muscle atrophy (Hirata et al., 2022). Additionally, interleukin 1 (IL-1) and IL-18 are also implicated in inflammation-mediated muscle atrophy (McBride et al., 2017; Dalle et al., 2017). While inflammation is a well-known contributor to muscle degradation, recent studies have begun to explore more specific inflammatory mechanisms such as pyroptosis, which may provide new insights into the molecular underpinnings of sarcopenia.

Pyroptosis involves apoptotic cells characterized by programmed cell death and is linked to inflammatory processes (Picca and Calvani, 2021). This phenomenon is typically initiated through two molecular pathways: one classical pathway mediated by Caspase-1 and another non-classical pathway involving Caspases 4/5/11, culminating in pyroptosis executed by members of the gasdermin protein family (Rosenberg, 1997; Ibebunjo et al., 2013; Rudolf et al., 2014). Both *in vivo* and *in vitro* investigations have indicated that activation of the NLRP3 inflammasome induces pyroptosis while promoting activation of the ubiquitin-proteasome system (UPS), resulting in muscle proteolysis and subsequent muscle atrophy (You et al., 2023). Factors related to inflammation and pyroptosis may serve as molecular markers for early diagnosis of muscular atrophy since their levels increase with age-related changes in muscle.

Currently, there are no predictive models based on the characteristics of IPRGs in musculoskeletal aging. Combining inflammation, pyroptosis, and immune infiltration analysis can more accurately identify diagnostic biomarkers. In this study, we systematically analyzed the expression of IPRGs, which play pivotal roles in inflammation-mediated muscle atrophy, along with their relationship to immune infiltration. We identified six key characteristic genes and constructed a diagnostic model, validated through RT-qPCR in a sarcopenia cell model, to support the early diagnosis of sarcopenia.

## Results

### DEGs between sarcopenia patients and healthy controls

**FIGURE 1:**
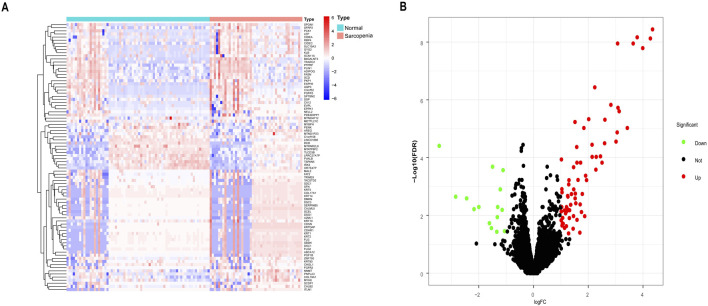
Differentially expressed genes (DEGs) between sarcopenia tissues and normal samples from the GSE111016 and GSE167186 datasets. **(A)** Heatmap of the top 50 DEGs. Colors range from red to blue, representing high to low expression levels of DEGs. **(B)** Volcano plot of DEGs. Red dots in the upper right denote upregulated DEGs, yellow dots in the upper left denote downregulated DEGs, and black dots in the middle represent genes with stable expression.

Using the “limma” package in R, DEGs were identified in a combined dataset of 44 elderly patients with sarcopenia and 68 normal controls. The analysis revealed 1,194 low-expressed genes and 2,196 highly expressed genes. The results of the DEGs were visualized using a volcano plot and heatmap (Figure 1). The top five most significantly upregulated genes were FLG2, FLG, KRT2, DSC1, and DSG1, while the top five most significantly downregulated genes were MTATP8P2, DCD, CHI3L1, PVALB, and MTRNR2L8. DEGs have been provided in the supplementary material (Supplementary Table 1).

### Functional enrichment analysis and GSEA

**FIGURE 2:**
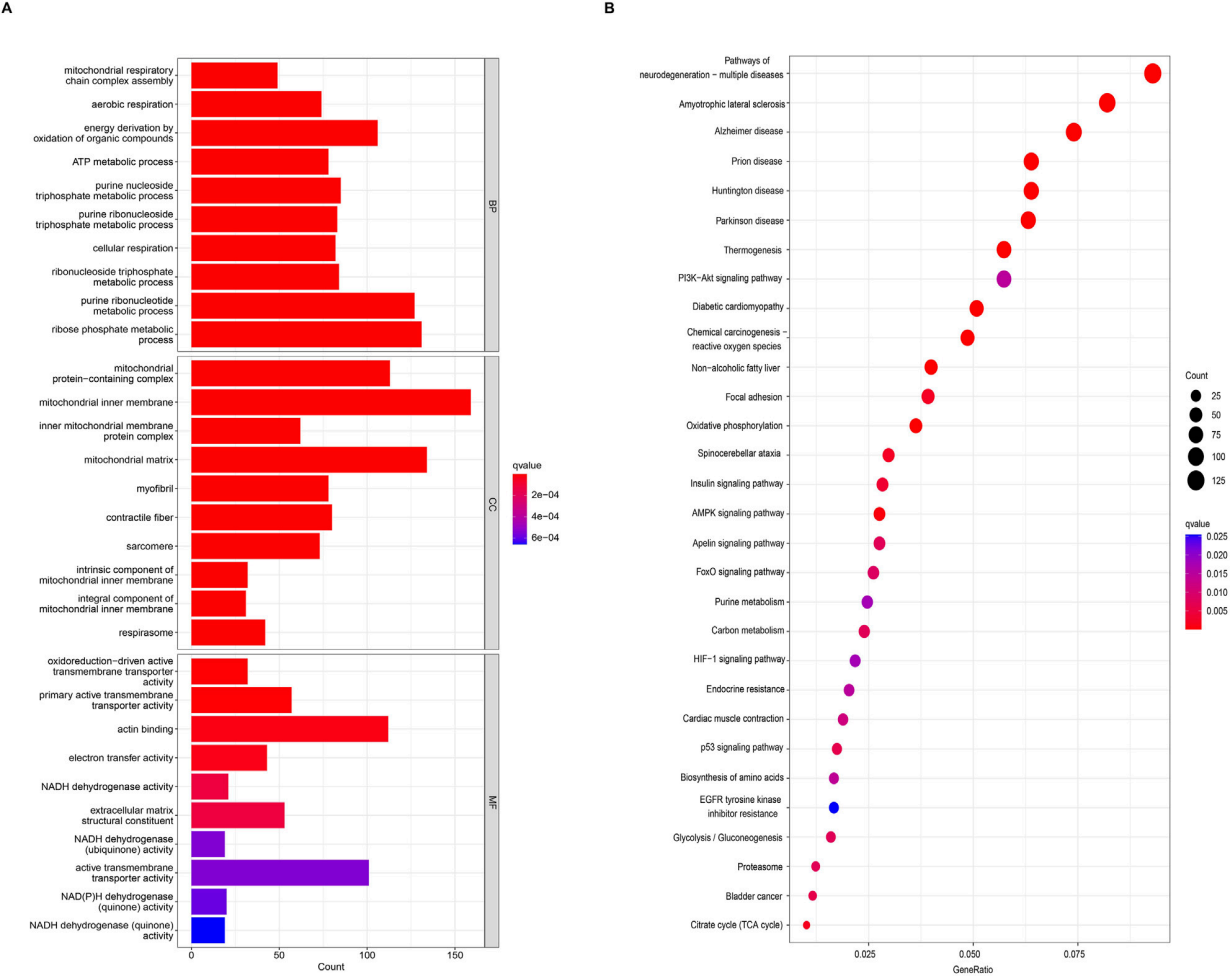
Functional and pathway enrichment analyses of DEGs. **(A)** Gene Ontology (GO) enrichment analysis of DEGs. **(B)** Kyoto Encyclopedia of Genes and Genomes (KEGG) enrichment analysis of DEGs.

**FIGURE 3:**
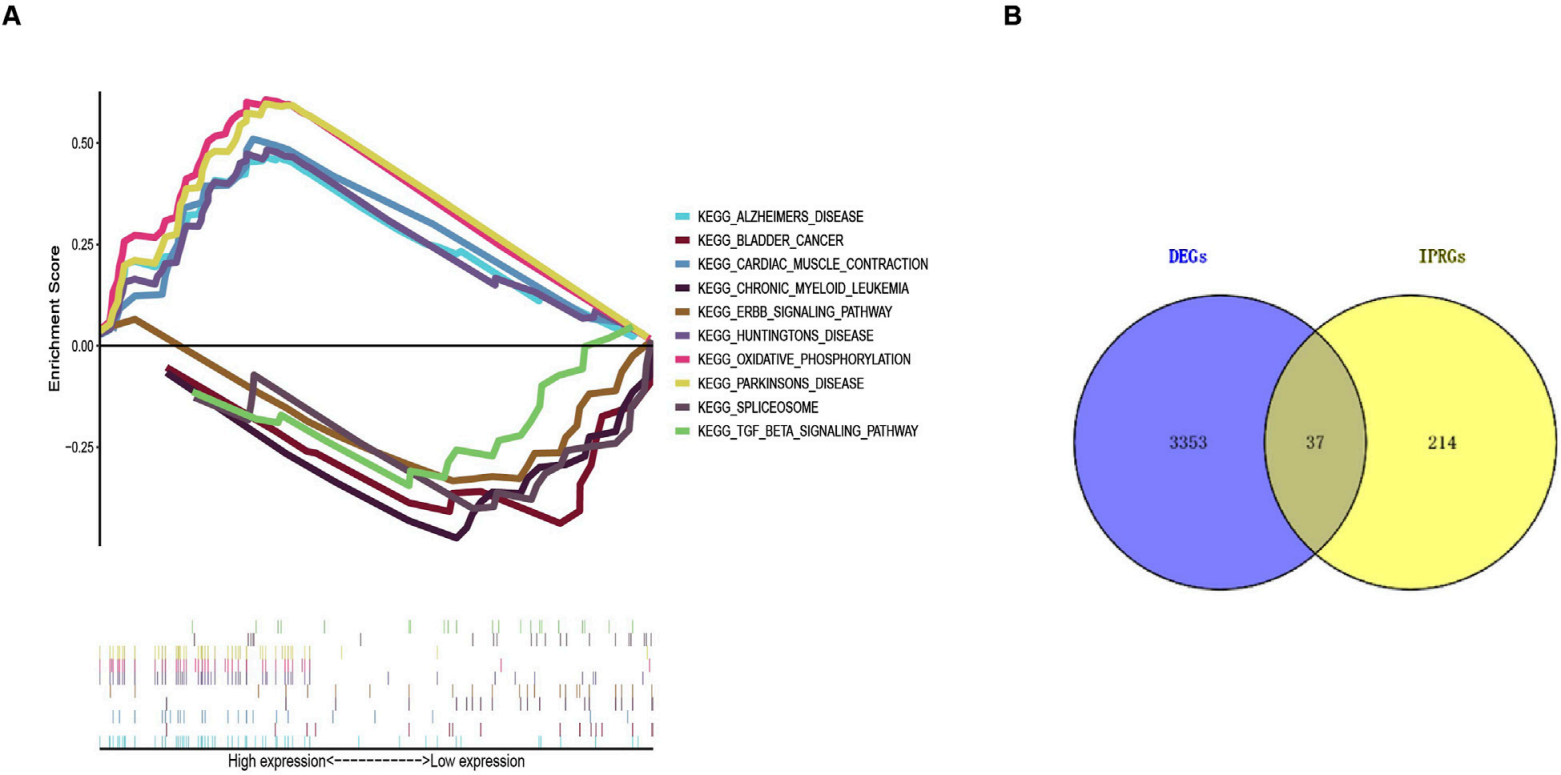
Gene Set Enrichment Analysis (GSEA) results and identification of IPRGs. **(A)** Enrichment results of the top five positively and negatively correlated pathways in sarcopenia. **(B)** Intersection of 3,390 DEGs with 251 IPRGs.

According to the screening criteria of an adjusted *p*-value <0.05, GO enrichment analysis of DEGs identified significant annotations across biological processes (BPs), cellular components (CCs), and molecular functions (MFs) (Figure 2A). BP analysis revealed that DEGs were primarily enriched in the ribose phosphate metabolic process, purine ribonucleotide metabolic process, energy derivation by oxidation of organic compounds, purine nucleoside triphosphate metabolic process, and ribonucleoside triphosphate metabolic process. CC analysis showed that DEGs were associated with the mitochondrial inner membrane, mitochondrial protein-containing complex, mitochondrial matrix, contractile fiber, and myofibril. MF analysis indicated that DEGs were enriched in actin binding, primary active transmembrane transporter activity, extracellular matrix structural constituent, oxidoreduction-driven active transmembrane transporter activity, and electron transfer activity. KEGG pathway enrichment analysis revealed that DEGs were mainly involved in pathways related to neurodegeneration-multiple diseases, amyotrophic lateral sclerosis, Alzheimer’s disease, prion disease, the PI3K-Akt signaling pathway, the AMPK signaling pathway, and the Foxo signaling pathway (Figure 2B). Furthermore, GSEA identified the top five gene sets most significantly enriched in both the sarcopenia and normal groups, suggesting that the development of musculoskeletal aging may be mediated by specific molecular mechanisms involving DEGs (Figure 3A).

### Identification of IPRGs and diagnostic biomarkers

**FIGURE 4:**
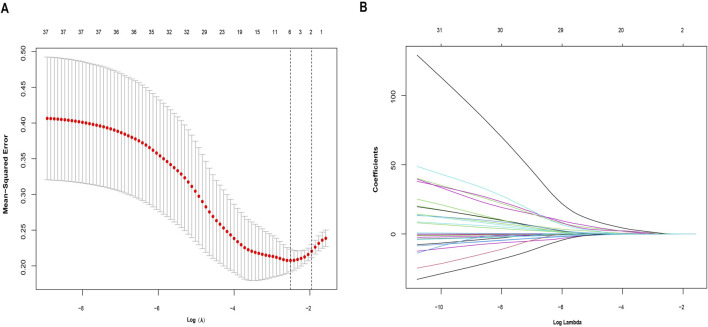
Identification of potential hub genes for sarcopenia using the LASSO regression model. **(A)** Selection of the optimal parameter for nonzero coefficients in the LASSO model. **(B)** Coefficient selection for the 6 hub genes based on LASSO analysis.

A collection of 251 IPRGs was acquired from the MSigDB database and PubMed. These genes intersected with the DEGs, resulting in 37 overlapping IPRGs, which were further examined. Figure 3B shows the 37 IPRGs in a Venn diagram. The expression profiles of 37 IPRGs were used to build the LASSO model. As shown in Figure 4A, the optimal λ value, which minimized classification errors, was determined. Based on this λ value, the LASSO coefficient spectrum of DEGs was analyzed (Figure 4B). Subsequently, 6 hub genes with nonzero coefficients were identified: GPC3, CYCS, FOXO3, SCN1B, AQP9, and BTG2.

### Development and validation of the model for sarcopenia

**FIGURE 5:**
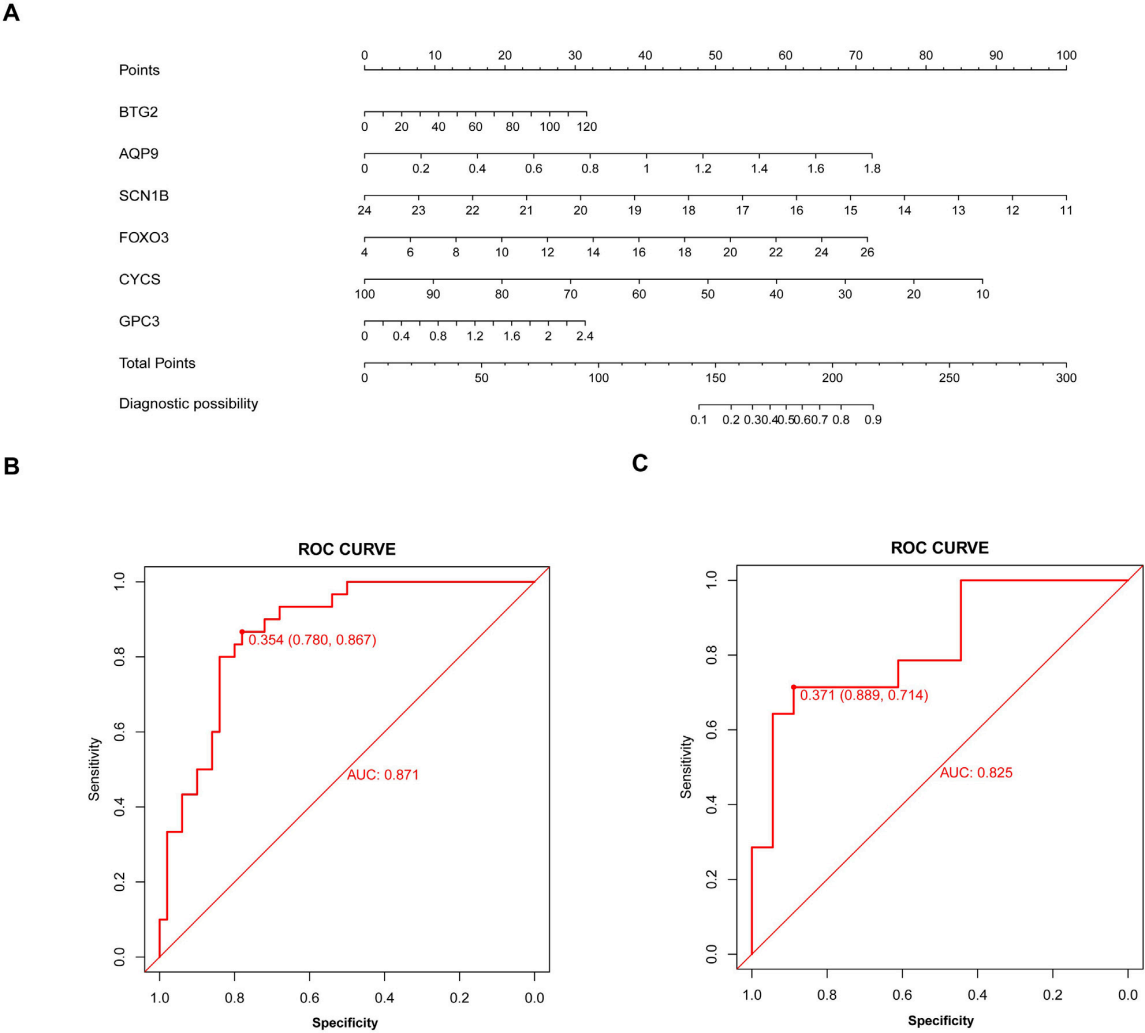
Nomogram model for sarcopenia. **(A)** Construction of the nomogram model based on six inflammation and pyroptosis-related genes (IPRGs). **(B)** Receiver operating characteristic (ROC) curve for the nomogram diagnostic model of sarcopenia. **(C)** ROC curve of the nomogram diagnostic model in the validation cohort.

**FIGURE 6:**
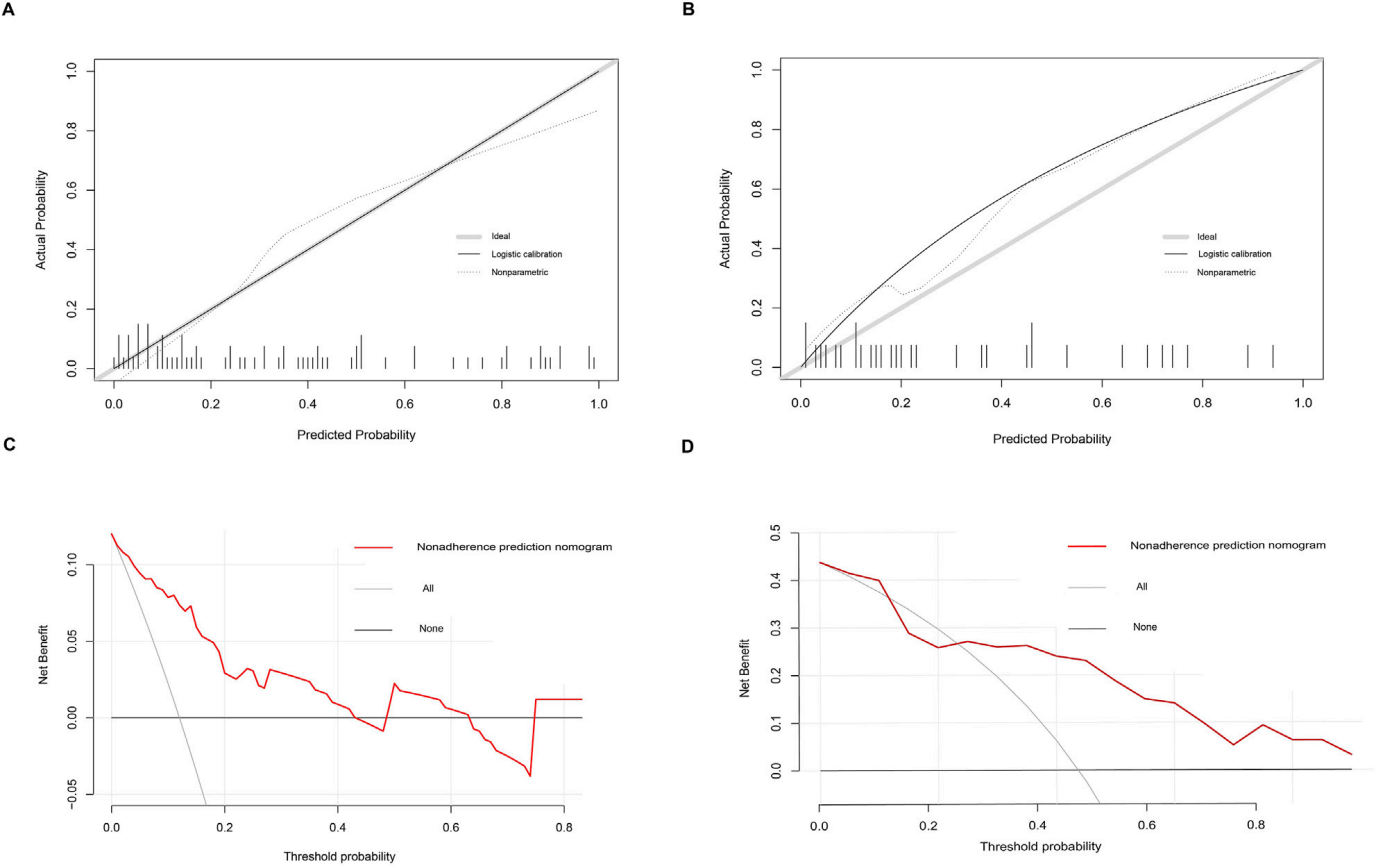
Calibration and decision curves for the nomogram model of sarcopenia. **(A, B)** Calibration curves showing the predictive ability of the nomogram model using the training and validation datasets. **(C, D)** Decision curve analysis (DCA) of the nomogram model using the training and validation datasets.

**FIGURE 7:**
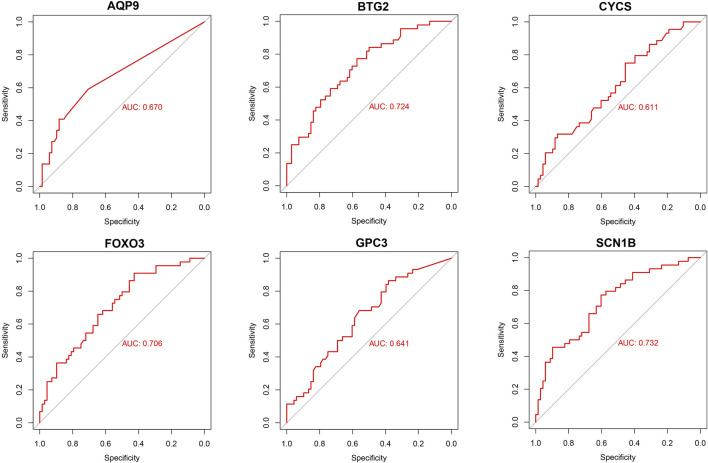
Validation of diagnostic validity for six diagnostic markers. ROC curves for GPC3, CYCS, FOXO3, SCN1B, AQP9, and BTG2 in the combined dataset.

Based on six diagnostic biomarkers, we developed a nomogram model to predict the onset of sarcopenia (Figure 5A). The model demonstrated promising performance, with an AUC of 0.871 on the training dataset (Figure 5B) and an AUC of 0.825 on the test dataset (Figure 5C). The high AUC values suggest that the model may serve as a valuable tool for early diagnosis of sarcopenia, potentially aiding clinicians in identifying at-risk patients before significant muscle loss occurs. These results indicate that our nomogram model exhibits high classification accuracy. Our study successfully constructed a diagnostic model for sarcopenia using the differential gene expression of these six biomarkers. The reliability of the nomogram model’s predictions is supported by the calibration curves (Figures 6A, B). Additionally, the Decision Curve Analysis (DCA) curve (Figures 6C, D) suggests that the model’s decisions may offer additional benefits for sarcopenia patients. We also plotted ROC curves for each of the six target genes individually (Figure 7). The results revealed that all six key genes had predicted AUCs >0.6, indicating their ability to successfully distinguish between sarcopenia and normal samples.

### Infiltration analysis of immune cells

**FIGURE 8:**
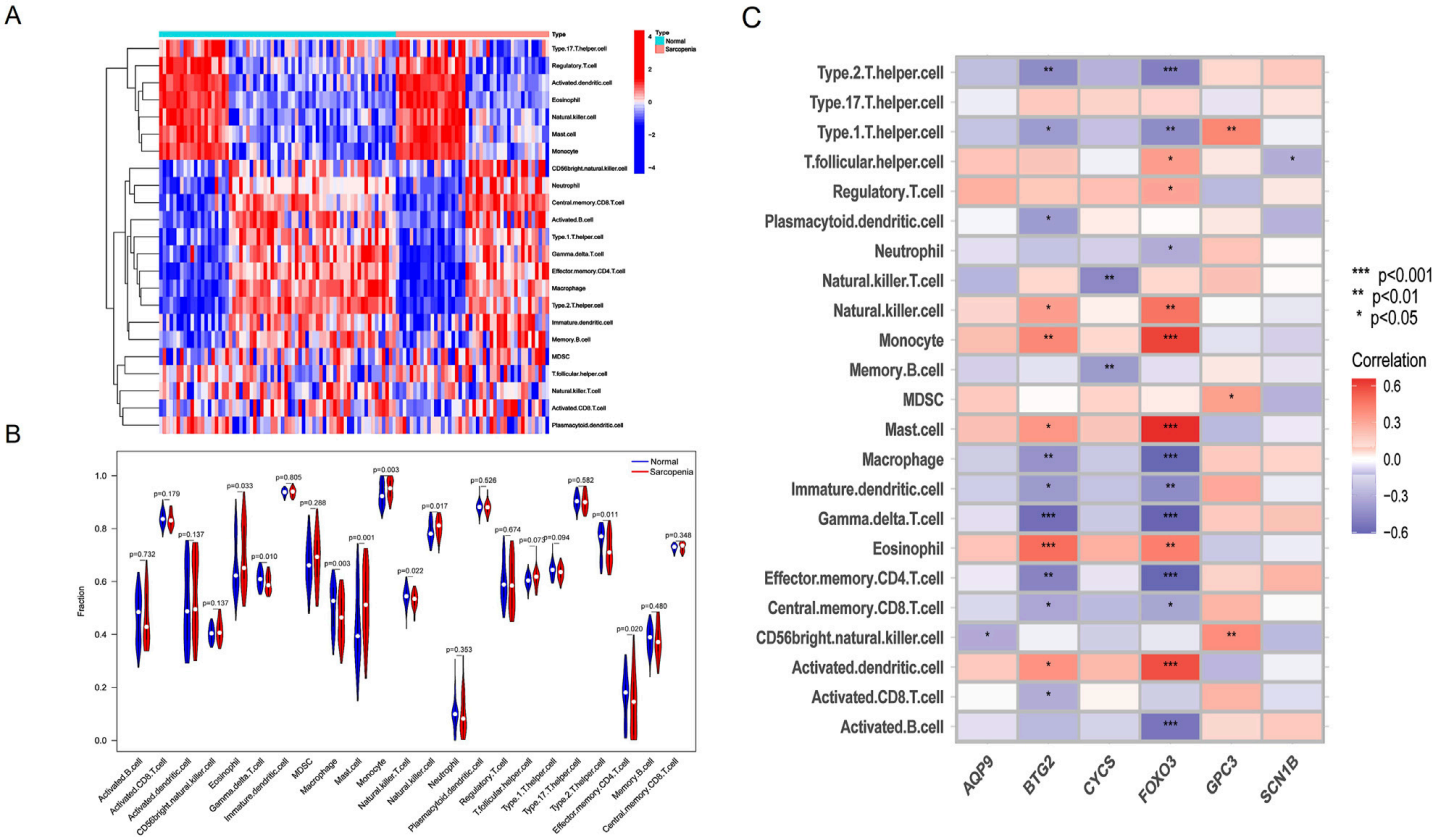
Immune cell infiltration differences between sarcopenia patients and healthy controls. **(A)** Heatmap showing the correlation between all DEGs and immune cells. **(B)** Violin plots comparing immune cell expression between normal and sarcopenia groups. **(C)** Correlation analysis between the six hub genes and immune cells.

To further investigate immune cell infiltration between sarcopenia patients and healthy controls, we used ssGSEA to evaluate the enrichment scores of different immune cell subsets. The results were visualized using a heatmap (Figure 8A) and violin plots (Figure 8B). Elevated levels of eosinophils, mast cells, monocytes, and natural killer cells were observed in sarcopenia patients, whereas levels of γδT cells, macrophages, natural killer T cells, and effector memory CD4 T cells were decreased. Additionally, we analyzed the association of six characteristic genes with immune cells (Figure 8C). Notably, FOXO3 showed a strong positive correlation with several immune cells, including monocytes, mast cells, and activated dendritic cells, and a strong negative correlation with other immune cells, including type 2 T helper cells, macrophages, γδT cells, and effector memory CD4 T cells.

### Validation of feature genes using RT-qPCR analysis

**FIGURE 9:**
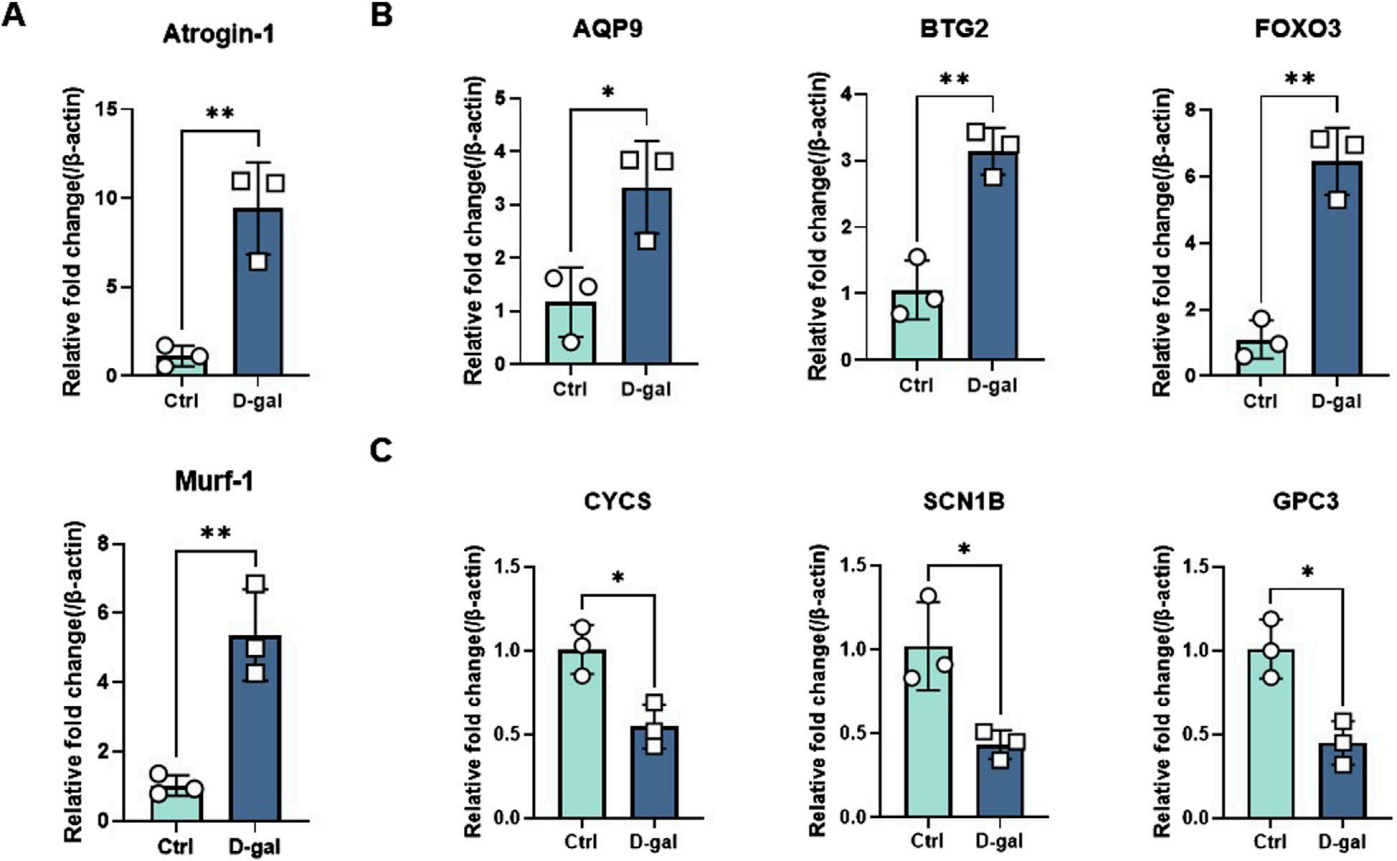
Evaluation of characteristic gene expression in muscle atrophy cells by RT-qPCR. **(A)** Marker genes for muscle atrophy. **(B, C)** Expression of elevated and decreased genes in sarcopenia cell models. Significant differences between groups were assessed using Student’s t-test. Data are presented as mean ± SD (*p < 0.05, **p < 0.01, ***p < 0.001, ****p < 0.0001).

To verify the expression of the six characteristic genes in sarcopenia, we constructed a muscle atrophy cell model. RT-qPCR results indicated that, compared to the control group, the muscle atrophy markers Atrogin-1 and Murf-1 were significantly upregulated, confirming the successful establishment of the muscle atrophy cell model (Figure 9A). In this model, RT-qPCR analysis revealed that three characteristic genes (BTG2, FOXO3, and AQP9) were highly expressed compared to the control (Figure 9B). Conversely, three other characteristic genes (GPC3, CYCS, and SCN1B) were significantly downregulated in the muscle atrophy cells relative to the control (Figure 9C).

## Discussion

Sarcopenia has emerged as a significant health concern due to an aging population; thus, early diagnosis of muscular atrophy has become imperative. In recent years, accurately predicting sarcopenia through screening relevant genes as diagnostic biomarkers has gained critical importance.

Although the pathogenesis of musculoskeletal aging is not fully understood, inflammation, pyroptosis, and immune infiltration in skeletal muscle cells are believed to play significant roles in its molecular mechanisms. Recent studies have indicated that the NLRP3 inflammasome and pyroptosis contribute to muscle dysfunction by reducing glycolytic potential and decreasing muscle fiber size (McBride et al., 2017). Additionally, chronic inflammation is linked to key characteristics of sarcopenia, such as increased skeletal muscle wasting, loss of strength, and functional impairment (Dalle et al., 2017; Picca and Calvani, 2021). Therefore, we hypothesize that inflammation and pyroptosis may be central mechanisms in musculoskeletal aging. To date, no studies have specifically explored the relationship between inflammation and pyroptosis in sarcopenia.

In this study, we developed and validated a predictive model for sarcopenia through bioinformatics analysis, which led to the identification of potential biomarkers. These findings are further supported by the confirmation of these biomarkers in follow-up experiments. Our study identified potential biomarkers for sarcopenia using LASSO analysis and experimental validation.

Firstly, we identified DEGs in the combined dataset through differential expression analysis. Next, we applied a LASSO regression model to 37 common genes related to inflammation and pyroptosis to screen for potential hub genes closely associated with the development of sarcopenia. GO enrichment analysis revealed that these DEGs are primarily involved in oxidative and metabolic processes within mitochondria. KEGG pathway enrichment analysis also indicated that these DEGs are strongly linked to pathways associated with various neurodegenerative diseases. Previous studies have shown that aging leads to gradual disorders in the neuromuscular system (Rosenberg, 1997). The neuromuscular junction (NMJ), a central component of this system, is a crucial synapse connecting motor nervous system excitability with skeletal muscle contraction (Ibebunjo et al., 2013; Rudolf et al., 2014; Punga and Ruegg, 2012). Recent research has increasingly highlighted the NMJ’s role in sarcopenia development (Gonzalez-Freire et al., 2014; Pannérec et al., 2016; Monti et al., 2021). On one hand, the NMJ is rich in mitochondria that supply the energy needed for neuromuscular transmission in the form of ATP (Anagnostou and Hepple, 2020; Zhou, 2021; Spendiff et al., 2016). On the other hand, mitochondria depend on PGC-1α as a cofactor for their transcriptional activities and normal metabolic functions (Austin and St-Pierre, 2012; St-Pierre et al., 2006). Furthermore, correlation analyses of the six hub genes with immune cells revealed strong associations with monocytes, macrophages, and mast cells, consistent with observations from DEGs and immune cell correlation analyses. In 2021, Afandy et al. found significantly higher MCP-1 levels in the sarcopenia group compared to the non-sarcopenia group (Afandy et al., 2021). MCP-1 promotes the migration and infiltration of monocytes to inflammation sites, contributing to muscle atrophy (Franceschi and Campisi, 2014; Bettcher et al., 2019; Curtis et al., 2015; Sell et al., 2006). Several studies have demonstrated that macrophages play a role in the regeneration and repair of aging-associated skeletal muscle (Sell et al., 2006; Sousa-Victor et al., 2015; Tidball, 2017; Fuchs and Blau, 2020; Zhang et al., 2020), exerting anti-inflammatory effects, clearing dead cells, and facilitating tissue repair through altered polarization states (Tidball, 2017; Fuchs and Blau, 2020). Additionally, a study revealed a significant increase in mast cells in the skeletal muscle of mice with malignant disease (Widner et al., 2021). These findings are consistent with our correlation analysis results.

The highlight of our study is the identification of six potential diagnostic markers for sarcopenia: BTG2, FOXO3, AQP9, SCN1B, CYCS, and GPC3.

Protein BTG2, also known as B-cell translocation gene 2, BTG family member 2, NGF-inducible anti-proliferative protein PC3, or NGF-inducible protein TIS21, plays a crucial role in regulating cell senescence, differentiation, and various other biological processes (Mauxion et al., 2009; Wheaton et al., 2010; Passeri et al., 2006). It can be induced by p53 to inhibit the cell cycle (Zhang et al., 2011), and its expression can be upregulated by stimuli such as IL-6 and growth factors (Yuniati et al., 2019). Previous studies have shown that miR-103-3p and miR-222-3p may influence the proliferation and differentiation of C2C12 myoblasts by targeting BTG2 (He et al., 2023; Yang et al., 2019). Peng et al. demonstrated that BTG2 could be a target for muscle aging by regulating MuSCs senescence (Peng et al., 2023). Our findings that BTG2 expression is significantly increased in the muscle atrophy group align with these studies, although the precise mechanism by which BTG2 regulates muscle atrophy requires further investigation.

FOXO family proteins, which are known for their highly conserved structural domains, are involved in crucial intracellular processes, including cell cycle regulation, oxidative stress response, inflammation, apoptosis, and energy metabolism (Dijkers et al., 2000a; Medema et al., 2000; Furukawa-Hibi et al., 2002; Kops et al., 2002; Ogg et al., 1997; Brunet et al., 1999; Dijkers et al., 2000b; Li et al., 2010). In mammals, the primary FOXO family members are FOXO1, FOXO3, FOXO4, and FOXO6 (Cao et al., 2023). Notably, FOXO3 is predominantly expressed in skeletal muscle (Curtis et al., 2015). Numerous studies have demonstrated that FOXO3 enhances the expression of atrogin-1 and MuRF1 through the IGF1-PI3K-AKT signaling pathway, leading to muscle atrophy (Sandri et al., 2004; Kamei et al., 2004; Southgate et al., 2009; Liu et al., 2007; Schiaffino et al., 2013). Additionally, activation of AMPK has been shown to promote muscle atrophy by increasing FOXO3 expression (Romanello et al., 2010; Nakashima and Yakabe, 2007; Sanchez et al., 2012). Interestingly, oxidative stress induction contributes to the activation of FOXO3 in models of disuse-mediated muscle atrophy (Suzuki et al., 2007; Piétri-Rouxel et al., 2010; Levine et al., 2008). It has also been reported that PGC-1α can work in conjunction with FOXO3 to mitigate muscle atrophy (Puigserver et al., 2003; Wu et al., 1999; Geng et al., 2011; Wenz et al., 2009). Aquaporins (AQPs) are a class of membrane channel proteins classified into water-selective channel proteins and aquaglyceroporins (Agre, 2004; Ishibashi et al., 1997; Ishibashi et al., 1998; Kishida et al., 2000). As a member of the latter group, AQP9 is primarily expressed in hepatocytes and plays a crucial role in gluconeogenesis and lipid metabolism by transporting glycerol (Trinchese et al., 2023; Inoue et al., 2009). Yang et al. first identified AQP9 expression in rat skeletal muscle in 2000 (Leek et al., 2012), with subsequent studies by Wang et al. and Inoue et al. confirming its presence in human skeletal muscle as well (Inoue et al., 2009; Yang et al., 2000; Wang et al., 2003). Although no reports have yet linked AQP9 directly to muscle atrophy, the skeletal muscle, as the largest endocrine organ in the body, also exhibits glycerol kinase activity (Inoue et al., 2009). Ren et al. suggested that the PI3K-AKT signaling pathway inhibits AQP9 (Ren and Wang, 2018), hinting at a potentially unexplored relationship between AQP9 and muscle atrophy that merits further investigation.

SCN1B, encoding the β1 and β1B subunits of voltage-gated sodium channels, is implicated in epilepsy and arrhythmia syndromes (O'Malley and Isom, 2015; Cervantes et al., 2022). It is widely recognized that muscle strength is influenced by both skeletal muscle and neurological factors (Arnold and Clark, 2023). The neuromuscular junction plays a critical role by converting electrical signals from presynaptic neurons into chemical signals that trigger muscle fiber contraction. Thus, SCN1B may impact muscle force production by regulating sodium influx and action potential generation. There is a clear positive relationship between SCN1B and muscle strength, consistent with our findings.

CYCS encodes cytochrome c, a mitochondrial membrane protein crucial for oxidative phosphorylation and apoptosis (Earnshaw, 1999; Liu et al., 1996; Kluck et al., 1997; Zou et al., 1997). Huang et al. demonstrated that overexpression of Mdfi (Myod family inhibitor) increased CYCS expression, promoting differentiation in C2C12 cells (Huang et al., 2021). Conversely, Kan et al. observed a significant decrease in CYCS expression with skeletal muscle aging (Kan et al., 2021). Baechler et al. identified that mitochondrial autophagy can activate CYCS to support myogenic differentiation (Baechler et al., 2019). These studies collectively highlight a strong association between CYCS and sarcopenia, although the underlying mechanisms warrant further investigation.

GPC3, a member of the glypican family, is typically expressed in embryonic tissues and various organs, and is notably overexpressed in hepatocellular carcinoma (Zhou et al., 2018; Wang et al., 2014; Xu et al., 2021; Qin et al., 2020). A study published in 2014 showed that GPC3 expression decreases with age in mouse skeletal muscle (Jones et al., 2014). However, our study did not find a significant difference in GPC3 expression between sarcopenia and control groups. This discrepancy may be due to the limited sample size; we plan to expand our sample and validate these results in future experiments and additional models.

In summary, our study developed and validated a risk-prediction model for sarcopenia, offering a precise biological tool for diagnosing sarcopenia in primary healthcare settings. By accurately identifying these potential biomarkers, the model aims to reduce instances of missed and misdiagnosed sarcopenia, thereby allowing for timely early intervention.

## Conclusion

In this study, we identified correlations between inflammation and pyroptosis-related genes, finding that 37 genes were differentially expressed in the sarcopenia group compared to controls. This indicates a significant interaction between inflammation and pyroptosis in the development of sarcopenia. Through bioinformatics analysis, we screened six IPRGs as potential diagnostic biomarkers for sarcopenia. We then developed a novel nomogram model based on these IPRGs, which demonstrated high diagnostic performance. The ROC curve analysis confirmed the significant predictive value of these biomarkers. Additionally, validation in a sarcopenia cell model supported the reliability of these six characteristic genes, reinforcing their potential as diagnostic biomarkers for sarcopenia.

## Materials and methods

### Data source and preprocessing

The gene expression profiles in the GSE111016 and GSE167186 datasets derived from bulk RNA sequencing were extracted from the public database GEO (http://www.ncbi.nlm.nih.gov/geo). The two raw datasets were transformed into an expression value matrix using the ‘limma’ package (Ritchie et al., 2015). Batch effects were removed using the “sva” package after merging the two datasets (Leek et al., 2012). The GSE111016 and GSE167186 datasets comprise 44 samples from patients with sarcopenia and 68 from healthy controls. All data were randomly divided into a 70% training dataset and a 30% validation dataset using R analysis software. The training dataset was used to create the screening model, while the validation dataset was used to verify the model’s performance.

### Identification of DEGs and IPRGs

DEGs were identified using the ‘limma’ R package with thresholds of |log2FC| > 0.1 and *p*-value <0.05. The results were visualized using volcano plots and heatmaps created with the “ggplot2” and “pheatmap” R packages. Inflammation-related genes (IRGs) were obtained from the HALLMARK_INFLAMMATORY_RESPONSE gene set in the Molecular Signature Database (MSigDB) (https://www.broadinstitute.org/msigdb) (Liberzon et al., 2015). Pyroptosis-related genes (PRGs) were collected from a previous study (Ye et al., 2021). After merging IRGs and PRGs, their intersection with DEGs was defined as IPRGs.

### Screening biomarkers and construction of the diagnostic nomogram model

Based on IPRGs, LASSO regression analysis was performed using R software package “glmnet” (Friedman et al., 2010) to identify key genes related to sarcopenia.

Based on the selected musculoskeletal aging candidate biomarkers, we used the R package ‘rms’ to predict the prevalence of sarcopenia and construct a new diagnostic nomogram model. This model is based on a logistic regression framework, where gene score assessments predict the probability of sarcopenia. Calibration curves were plotted to evaluate the consistency between predicted and actual values. Additionally, Decision Curve Analysis (DCA) was performed to assess the clinical benefit of the nomogram model’s decisions for patients.

### Evaluation and verification of nomogram model

The ROC curve was generated using the R package “pROC”, and the AUC was calculated to evaluate the diagnostic performance of the novel model. Additionally, the AUC and confidence interval (CI) were used to validate the model’s efficiency.

### Functional enrichment analysis and GSEA

To further elucidate the characteristic biological properties of DEGs, we conducted functional enrichment analysis using the “clusterProfiler” package in R (Yu et al., 2012) This analysis included Gene Ontology (GO) and Kyoto Encyclopedia of Genes and Genomes (KEGG) pathway analyses. GO terms were categorized into three categories: molecular function (MF), biological process (BP), and cellular component (CC). Items with a corrected *p*-value <0.05 were considered significantly enriched among the candidate genes (CGs). Bubble plots and bar charts were created using the “ggplot2” and “enrichplot” packages in R to visualize the KEGG enrichment analysis of CGs. GSEA is a computational method used to determine whether predefined gene sets exhibit statistical differences between two biological states. It is commonly employed to assess changes in expression, biological processes, pathways, and activities within dataset samples (Subramanian et al., 2005). To investigate differences in biological processes between the two sample groups, enrichment analysis and visualization were performed using the GSEA method in the “clusterProfiler” package in R, based on the gene expression profile dataset. Adjusted *p*-values <0.05 were considered statistically significant.

### Infiltration analysis of immune cells

The infiltration scores of immune cells and the activity of immune functions in the healthy and sarcopenia groups were calculated using single-sample Gene Set Enrichment Analysis (ssGSEA) with the “gsva” R package (Park et al., 2019). These results were visualized using heatmaps generated with the “pheatmap” package (Dailey, 2017). To compare and visualize the proportion of infiltrating immune cells between healthy and sarcopenia samples, violin plots were created using the ‘ggpubr’ R package (Hu, 2020).

### Construction of muscular atrophy cell model

Murine C2C12 myoblasts, obtained from ATCC, were cultured at 37°C with 5% CO2 in DMEM supplemented with 80 U/mL penicillin, 0.08 mg/mL streptomycin, and 10% fetal bovine serum (Gibco, United States). To induce differentiation into myotubes, sub-confluent myoblasts were transferred to DMEM containing 2% horse serum (Biological Industries, Israel) and cultured for 4–6 days (Wang et al., 2021). To construct a cellular model of sarcopenia, mature myotubes were treated with varying concentrations of D-gal (20 g/L, Sigma, United States) for 24 h (Yang et al., 2021).

### Real-time fluorescence quantitative PCR

Total RNA was extracted from C2C12 myotube samples using the Total RNA Extraction Kit (TIANGEN, China). Complementary DNA (cDNA) synthesis was carried out with the PrimeScript™ RT reagent Kit (Perfect Real Time) (Takara, Japan). Quantitative real-time PCR was conducted with Maxima SYBR Green/ROX qPCR Master Mix (2X) (Thermo Scientific, United States) on the LightCycler480 system (LightCycler, United States). Relative gene expression levels were determined by the 2^−ΔΔCT^ method, using beta-actin (β-actin) as the internal control. Primer sequences are listed in Supplementary Table S1.

## Statistical analysis

The statistical analysis was performed using R software (version4.2.0) and GraphPad Prism (Version 9.0). Continuous variables were expressed as mean ± SD or median (quartile). The Student’s t-test and Mann–Whitney test were used to compare continuous variables with and without normal distribution, respectively. Categorical variables were presented as counts (percentages) and analyzed using the chi-square test. All statistical *p*-values were two-sided, with *p* < 0.05 considered statistically significant.

## Data Availability

The original contributions presented in the study are included in the article/Supplementary Material, further inquiries can be directed to the corresponding authors.
