# Supplementary material for: Predictive models of sarcopenia based on inflammation and pyroptosis-related genes
Source: Front Genet. 2024 Dec 24;15:1491577. doi: 10.3389/fgene.2024.1491577 (PMC11703911; doi:10.3389/fgene.2024.1491577)
Supplement: Supplementary file 3 [file Table1.docx]

**Table S1. Primer sets for Real-time PCR analyses**

| Gene | Forward primer (5′ to 3′) | Reverse Primer (5′ to 3′) |
| --- | --- | --- |
| β-actin | AGCCTCGCCTTTGCCG | CTCGTCGCCCACATAGGAAT |
| Atrogin-1 | CAGCTTCGTGAGCGACCTC | GGCAGTCGAGAAGTCCAGTC |
| MuRF1 | GTGTGAGGTGCCTACTTGCTC | GCTCAGTCTTCTGTCCTTGGA |
| SCN1B | GGATTCCGATACCGAGGCTG | GGTCCACTCCGTGAAGGTTT |
| FOXO3 | CTGGGGGAACCTGTCCTATG | TCATTCTGAACGCGCATGAAG |
| GPC3 | CAGCCCGGACTCAAATGGG | CAGCCGTGCTGTTAGTTGGTA |
| AQP9 | TGGTGTCTACCATGTTCCTCC | AACCAGAGTTGAGTCCGAGAG |
| BTG2 | ATGAGCCACGGGAAGAGAAC | GCCCTACTGAAAACCTTGAGTC |
| CYCS | CCAAATCTCCACGGTCTGTTC | ATCAGGGTATCCTCTCCCCAG |
